# OCCASIONAL DIGESTIVE HEMORRHAGE IN CHILDREN DUE TO STRONGYLOIDIASIS: IMPORTANCE OF PARASITOLOGIC TESTING

**DOI:** 10.1590/1984-0462/;2019;37;1;00013

**Published:** 2018-07-26

**Authors:** Evandro Brandelero, Bibiana Paula Dambrós, Elenice Messias do Nascimento Gonçalves, Vera Lucia Pagliusi Castilho, Amarildo Moro Ribas, Maribel Emília Gaio

**Affiliations:** a Laboratório de Análises Clínicas Vida EireliEpp, Videira, SC, Brasil.; b Universidade do Oeste de Santa Catarina, Videira, SC, Brasil.; c Universidade de São Paulo, São Paulo, SP, Brasil.; d Consultório de Pediatria, Videira, SC, Brasil.

**Keywords:** Strongyloidiasis, Superinfection, Strongyloides stercoralis, Immunosuppression, Estrongiloidíase, Superinfecção, Strongyloides stercoralis, Imunossupressão

## Abstract

**Objective:**

To describe an uncommon case of infection by *Strongyloides stercoralis* (*S. stercoralis*) in a 4-month-old child and to highlight the importance of early diagnosis.

**Case description:**

The patient was a male child from the city of Videira, State of Santa Catarina, Southern Brazil, who was born preterm by Cesarean-section, weighing 1,655 g, and stayed in the neonatal intensive care unit for 20 days. At four months of age, the child started presenting blood in stools and the possibility of cow’s milk protein allergy was considered, given the symptoms and the use of infant formula in his 1^st^ semester of life, which was then replaced by infant formula with hydrolyzed protein. White blood cell count and a parasitological stool sample were requested. Both tested positive and the stool ova and parasite examination showed a rhabditoid larva of *S. stercoralis*. The clinician maintained the initial hypothesis and diet, but requested three new stool samples, which tested positive for rhabditoid larvae of *S. stercoralis*. Since the child presented abdominal pain and vomiting, and there was still blood in stools, treatment with thiabendazole was initiated twice a day for two days. Treatment was repeated after seven days along with a new parasitological examination, which was then negative.

**Comments:**

Although strongyloidiasis is usually a mild parasitic infection, it may be severe and disseminated in immunocompromised patients. This agent must be considered in patients who live in endemic areas, and the diagnosis should be established by searching *S. stercoralis* larvae in tracheal secretions and in stools.

## RESUMO

**Objetivo:**

Descrever um caso incomum de infecção por *Strongyloides stercoralis* (*S. stercoralis*) em paciente de quatro meses de idade e ressaltar a importância do diagnóstico precoce.

**Descrição do caso:**

Paciente masculino, procedente e residente de Videira, Santa Catarina, Brasil, nasceu pré-termo, parto cesárea, peso de nascimento 1.655 g, e permaneceu na Unidade de Terapia Intensiva neonatal e intermediária por 20 dias. Aos quatro meses de idade, começou a evacuar fezes sanguinolentas e foi feita hipótese de alergia à proteína do leite de vaca, em razão da sintomatologia e do uso da fórmula infantil para o primeiro semestre, para o qual foi indicada a substituição por fórmula infantil com proteína hidrolisada. Foram solicitados a pesquisa de leucócitos e o exame parasitológico das fezes). Ambos se mostraram positivos e o parasitológico revelou a presença de larva rabditoide de *S. stercoralis*. O clínico manteve a hipótese inicial e a dieta, mas solicitou a coleta de três amostras de fezes, que resultaram em uma amostra para larvas rabditoide, em muda, de *S. stercoralis*. Como a criança apresentava dor abdominal, vômito e as fezes permaneciam sanguinolentas, foi iniciado o tratamento com tiabendazol - duas vezes/dia por dois dias -, repetido após sete dias, e, em seguida, realizado o exame parasitológico de fezes, tendo sido negativo.

**Comentários:**

A estrongiloidíase, apesar de ser uma infecção parasitária frequentemente leve, em pacientes imunocomprometidos pode se apresentar de forma grave e disseminada. Deve-se suspeitar desse agente em pacientes que vivem em áreas endêmicas, sendo o diagnóstico estabelecido por meio da pesquisa das larvas do *S. stercoralis* na secreção traqueal e nas fezes.

## INTRODUCTION

Strongyloidiasis is considered a neglected tropical disease caused by the intestinal nematode *Strongyloides stercoralis* (*S. stercoralis*) and characterized by gastrointestinal and/or pulmonary involvement.^1^ Approximately 25% of the world population is estimated to be infected with intestinal helminths^2^, with strongyloidiasis affecting 100 million people worldwide^1^ and being responsible for a high endemicity in Latin America.^3^ Reports of the prevalence of intestinal parasitic diseases in Brazil are specific and have been described in different populations,^4^ varying from 3 to 82%.^5^ A study conducted in the metropolitan area of Rio de Janeiro from April 2012 to February 2015, with 3,245 individuals of both genders, reported 4.3% of infections caused by *S. stercoralis*.^6^

According to Rey,^7^ the most common form of contamination is active skin penetration by the filarioid larva. Another less common form of contamination is by the digestive tract, through ingestion of water contaminated with infecting larvae. According to Albarqi et al.,^8^ once the larvae penetrate the skin, they reach the bloodstream and invade the lungs’ alveoli; this pulmonary migration may cause pneumonia, but usually asymptomatic. The larvae are then expectorated, traveling through the trachea and then swallowed. The larvae mature and become adult parthenogenic females, which release eggs into the gastrointestinal tract. The eggs hatch while still in the gastrointestinal tract and give rise to rhabditoid larvae, which are excreted. However, some of these larvae become infectious (filarioid) and penetrate the anal mucosa and perianal skin, re-entering the circulatory system and restarting the cycle. Because of this autoinfection cycle, a person can be infected with *S. stercoralis* for decades.

The infection is usually asymptomatic but may bring about a combination of uncertain clinical symptoms such as: severe epigastric pain, chronic diarrhea, constipation, indigestion, anorexia, anal pruritus, abdominal distension, weight loss, nausea, vomiting, peripheral eosinophilia, asthenia, adynamia, fever, hemorrhage, anemia and, rarely, obstruction of the small intestine.^9^ ^,^ ^10^

The objective of this paper was to describe an unusual case of *S. stercoralis* infection in a four-month-old infant and to highlight the importance of diagnosis and specific treatment. The study was approved by the Research Ethics Committee of Universidade do Oeste de Santa Catarina and Hospital Universitário Santa Terezinha (UNOESC/HUST) under CAAE 66365617.6.0000.5367 and approval opinion n. 2,032,547.

## CASE REPORT

Male patient, resident and born in the city of Videira, Santa Catarina, Brazil, with a small interatrial communication with no hemodynamic repercussion. Born by Cesarean-section, at 33 weeks of gestational age, weighing 1,655 g and cephalic perimeter measuring 29.5 cm, with 1^st^ and 5^th^ minute APGAR Bulletins of 7 and 8, respectively. At birth, he presented respiratory distress syndrome and some episodes of apnea, being then referred to the neonatal intensive care unit (NICU) for seven days, followed by 13 days in intermediate care. He was put to an oxygen hood, without mechanical ventilation, and received antibiotic therapy with ampicillin and gentamicin for ten days. Then he underwent phototherapy after developing neonatal jaundice and evolved well. Ophthalmic, audiometric and neurological evaluations were normal. Twenty-five days after hospital admission, and weighing 2,090 g, the patient was discharged with indication of periodic consultations with a clinician to monitor his development. His diet, from birth, comprised breast milk supplemented with infant formula for the first six months of his life.

**Figure 1:**
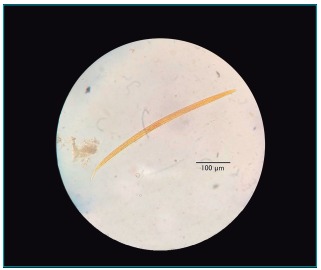
Rhabditoid larva of *Strongyloides stercoralis* isolated from the second stool sample collected from the infant.

At 4 months of age, weighing 5,570 g and measuring 59 cm, the patient began to evacuate bloody stools, and the clinician raised the hypothesis of cow’s milk protein allergy due to the symptomatology and supplementation to initial breastfeeding, and replaced it with infant formula with hydrolyzed protein. Fecal culture, fecal leukocyte test and ova and parasite (O&P) examination were requested. The fecal culture tested negative and fecal leukocyte test was positive. As for O&P examination, performed by the Hoffman-Pons-Janer method (Lutz),^11^ the test was positive for rhabditoid larvae of *S. stercoralis*. The clinician maintained the initial hypothesis of cow’s milk protein allergy and the protein-free diet, but requested three new stool samples for O&P examination; two of them tested negative and one was again positive for rhabditoid larvae in *S. stercoralis* (Figure 1). The following morphological features were found in the parasite: short esophagus, with presence of a “mass” in posterior portion, similar to the esophageal bulb; the center of the body was a darker region, in which a small structure resembling the genital primordium and an apparently non-pointed tail could be seen (Figure 1).

**Figure 2:**
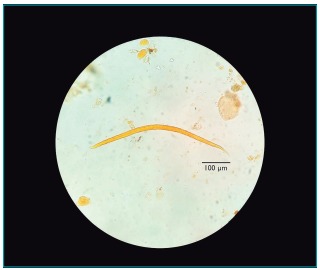
Filarioid larva of *Strongyloides stercoralis* isolated from the second stool sample collected from the father of the infant.

With positive parasitological result confirmed and the child still in abdominal pain accompanied by vomiting and bloody stools, treatment with thiabendazole at the dose of 50 mg/kg/day, split in two daily doses for two days, was started. After 48 hours, the child was calmer, less disturbed by pain and apparently without blood in stool. After seven days, the initial therapeutics was repeated and monitoring was made with three new stool samples collected on alternate days and submitted to O&P examination. All results were negative. Although the infant had gained weight after the drug treatment, the clinician maintained breastfeeding associated with enteral or oral infant formula with hydrolyzed proteins due to the cow’s milk protein allergy hypothesis, with subsequent reintroduction of normal protein for verification. Recommendation was that the parents, sister and caregiver of the child should be submitted to O&P examination, with three serial stool samples. All samples tested negative, except for the second sample of the father, which was positive for filarioid larvae of *S. stercoralis* (Figure 2), as it presented long esophagus, with visible bowel junction and non-pointed tail. This diagnosis led to the introduction of treatment with single-dose ivermectin for all. Subsequently, a control O&P examination was performed. All relatives then tested negative.

## DISCUSSION

The symptom presented by the patient described in this study was bloody stools. According to Hizal et al.,^12^ digestive hemorrhage is a serious entity in children, because it can quickly debilitate the patient, even when blood loss is mild. The clinical presentation and age of patients should be taken into account for differential diagnosis of low gastrointestinal bleeding, since this population constitutes approximately 0.3% of hospitalizations in pediatric emergency units, but only 4% of cases are associated with conditions posing threat to patients’ life.^13^

The hypothesis raised by the physician was cow’s milk protein allergy. According to studies in children, this condition has blood and mucus in stools among its gastrointestinal manifestations.^14^ ^,^ ^15^ However, the correct diagnosis based on oral tolerance tests for appropriate treatment establishment should be considered.^14^

O&P examination confirmed the presence of rhabditoid larvae of S*. stercoralis*. According to Mejia et al.,^16^ in individuals infected by *S. stercoralis,* acute strongyloidiasis may manifest as skin irritation on the spot of larvae penetration, followed by tracheal irritation or dry cough and, lastly, gastrointestinal symptoms such as diarrhea, constipation, abdominal pain or anorexia. In addition, this parasitosis may be chronic, presenting as an asymptomatic infection and/or, as in most times, only with mild gastrointestinal symptoms (diarrhea, constipation or intermittent vomiting).^17^ According to Geri et al.,^18^ gastrointestinal symptoms are common clinical manifestations in 71.2% of patients. According to Rios et al.,^19^ the hyperinfection syndrome is frequently associated with the administration of corticoids and other immunosuppressive conditions. Paredes et al.^13^ state that this parasitosis can relate to primary and secondary morbidities due to underestimated digestive bleeds.

O&P examination is the main tool to identify a wide variety of enteric parasites, including protozoa and helminths. It is indicated for patients with gastrointestinal disorders such as bloody diarrhea, eosinophilia, infections by enteric organisms, exposure to endemic areas, or those belonging to risk groups: children, the elderly, immunosuppressed or hospitalized patients, and health professionals. Those who present eosinophilia should also undergo microscopic evaluation of other biological materials according to symptoms, that is, sputum, bronchoalveolar lavage, duodenal juice, urine, cerebrospinal fluid, ocular secretion, biopsies, among others.^20^

Regarding O&P examination, the stool samples collected in this study were submitted to the Hoffman-Pons-Janer method (Lutz),^11^ and from four samples collected from the infant, two were positive. This corroborates the findings by Sudré et al.,^5^ who reported that a single stool sample searched for larvae is able to detect about 30% of infections, and diagnosis sensitivity increases to about 50% if three samples are used, reaching close to 100% with the use of seven samples. Sensitivity optimization, in this case, could have been achieved with associated use of extraction technique based on positive hydro-thermo tropism found in larvae, like the method proposed by Baermann & Moraes,^21^ with agar plate^22^ or filter paper stool culture.^23^

Regarding the quality of samples, according to the laboratory, the infant’s stools were reduced in quantity, dried and collected from the diaper. Studies state that fresh stool samples should be sent to the laboratory within 30 minutes after collection for process optimization, although it is not viable in most situations. For this reason, preservatives in proportion 3:1 are recommended to allow late examination of samples.^24^

Like adult parasites, filarioid larvae are rarely seen in feces, except in cases of hyperinfection and intestinal constipation. Some rhabditoid larvae may spontaneously turn into filarioid larvae in unfixed stool samples stored at room temperature for a few hours. Larvae that have been dead for many days have their structured modified, which also makes diagnosis difficult.^5^ Thus, it is believed that the time between sample collection and its processing may have incurred the rhabditoid larvae into molt.

According to review carried out by Geri et al.,^18^ the standard anthelmintic treatment for strongyloidiasis includes thiabendazole in 35.3%, albendazole in 28.9%, and ivermectin in 40,6% of cases. Mejia et al.,^16^ also reported that recommended drugs are oral ivermectin for two days; oral albendazole twice daily for three days, or single-dose thiabendazole. These protocols reinforce the correct standard prescription of the anti-helminthic agent by the physician.

This case tells us that *S. stercoralis* research should be included in the differential diagnosis of gastrointestinal bleeding, especially in populations considered at risk, such as newborns and infants, due to their immunological and physiological immaturity, situation of hospitalization, being in contact with health professionals, and residing in endemic areas.

At that, the level of suspicion for diagnosis of parasitosis should be increased, considering that intestinal parasitic infections are still common in Brazil. Clinical laboratories are key in providing accurate results and identifying parasites, thus swiftly and effectively guiding the medical decision. Efforts should be directed to establishing preventive measures through health education, including personal and home hygiene, care of infants and relatives, and strongyloidiasis control and investigation. Guided treatment should be encouraged, and only in specific cases should the empirical treatment of parasites be carried out.
